# Innate Non-Specific Cell Substratum Adhesion

**DOI:** 10.1371/journal.pone.0042033

**Published:** 2012-08-31

**Authors:** William F. Loomis, Danny Fuller, Edgar Gutierrez, Alex Groisman, Wouter-Jan Rappel

**Affiliations:** 1 Section of Cell and Developmental Biology, Division of Biological Sciences, University of California San Diego, La Jolla, California, United States of America; 2 Department of Physics, University of California San Diego, La Jolla, California, United States of America; 3 Center for Theoretical Biological Physics, University of California San Diego, La Jolla, California, United States of America; Cardiff University, United Kingdom

## Abstract

Adhesion of motile cells to solid surfaces is necessary to transmit forces required for propulsion. Unlike mammalian cells, *Dictyostelium* cells do not make integrin mediated focal adhesions. Nevertheless, they can move rapidly on both hydrophobic and hydrophilic surfaces. We have found that adhesion to such surfaces can be inhibited by addition of sugars or amino acids to the buffer. Treating whole cells with αlpha-mannosidase to cleave surface oligosaccharides also reduces adhesion. The results indicate that adhesion of these cells is mediated by van der Waals attraction of their surface glycoproteins to the underlying substratum. Since glycoproteins are prevalent components of the surface of most cells, innate adhesion may be a common cellular property that has been overlooked.

## Introduction

Motile cells require traction for translocation on surfaces. For many mammalian cell types binding of specific surface receptors to components of the extracellular matrices is thought to provide the necessary traction. Focal adhesions form where integrin heterodimers bind to matrix components on the outside and associate with F-actin and other cytoskeletal proteins on the inside [1], [2]. These complexes provide strong, relatively stable, adhesion of the cell to the matrix. However, mouse leukocytes which have been genetically engineered to lack integrins are able to move through collagen matrices in the absence of focal adhesions [3], [4]. Likewise, treating polymorphonuclear leukocytes (PMN) with antibodies to bετα1, bετα2 and alphaV bετα3 integrins did not affect chemotaxis on glass coverslips in the presence of human serum albumin [5]. It appears that the innate adhesion of these cells is sufficient to provide traction.

The highly motile cells of the social amoeba *Dictyostelium discoideum* cannot form integrin mediated focal adhesions because they do not carry genes encoding integrin homologs in their genome [6], [7]. Moreover, they do not have genes encoding the major extracellular matrix components such as fibronectin, collagen, fibrin, laminin, or vitronectin. When *Dictyostelium* cells that are growing exponentially in suspension are washed and deposited on clean glass or plastic, they attach and start to move within a few minutes showing that they can get traction without any need to deposit extracellular matrix material [8]–[10]. Furthermore, cells developing in microfluidic devices translocate for long distances over untreated glass where the flow would sweep away secreted materials 11–13. It appears that these cells can form substrate adhesions in the absence of receptors for specific ligands in extracellular matrices.

*Dictyostelium* cells move equally fast on the hydrophobic surface of freshly cleaved mica as they do on borosilicate glass or the hydrophilic surface of glass coated with bovine serum albumin (BSA) [10]. It appears they can gain traction on both hydrophilic and hydrophobic surfaces equally well. Using a radial flow detachment assay, Decave et al., [14] were able to quantitate adhesion of *Dictyostelium* cells to untreated glass and glass with a hydrophobic coating. Cells were dislodged in a first order manner at a rate that depended on the shear stress. Although the cells were slightly more adherent to the coated glass, they adhered well to both substrates further arguing against specific hydrophilic or hydrophobic interactions playing significant roles in substratum adhesion under these conditions.

If dedicated adhesion proteins are not involved in substratum adhesion, how can the cells stick to both hydrophilic and hydrophobic substrates and translocate well? Perhaps the molecular surface of cells is such that adhesive forces can be generated other than by ligand binding or ionic interaction. One possibility is van der Waals attraction between the surface of the cell and the substratum. Van der Waals attraction arises from the interaction between permanent or induced dipoles and, although of varying strengths, can be significant. In the case of a cell attached to a substratum, it is useful to consider both the cell membrane and the substrate as an infinite slab, separated by a distance *l*. Then, the force per unit area between the cell and the substrate is approximated by 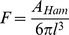 where *A_Ham_* is the Hamaker coefficient [15], [16]. This coefficient is a function of the dielectric constant and the polarizability of the substrate, the cell membrane and the medium. It has been calculated by Nir and Andersen [17] for a number of realistic cell-substrate cases and was found to be in the range of 1–10×10^−21^J. To calculate the magnitude of van der Waals attraction forces between the cell and the substratum, we need an estimate of both the contact area of the cell-substrate interface and the distance between the cell membrane and the substratum. Considering a circular cell-substratum interface with a radius of 5 µm that is 30 nm away from the substratum [18], we can estimate the van der Waals attraction force to be ∼100–1000 pN. This is comparable with traction force microscopy measurements which found that the maximum contraction force generated by *Dictyostelium* cells was ∼200 pN [19]. The resulting forces are also consistent with the forces required to dislodge cells from untreated glass [14]. Importantly, small molecules dissolved in the medium that have chemical determinants similar to those in the cell membrane can significantly reduce the van der Waals attraction forces [17]. We have explored the effects of adding sugars and amino acids to the buffer used in a microfluidic based adhesion assay of cells deposited on both hydrophilic and hydrophobic substrates.

## Results

**Figure 1:**
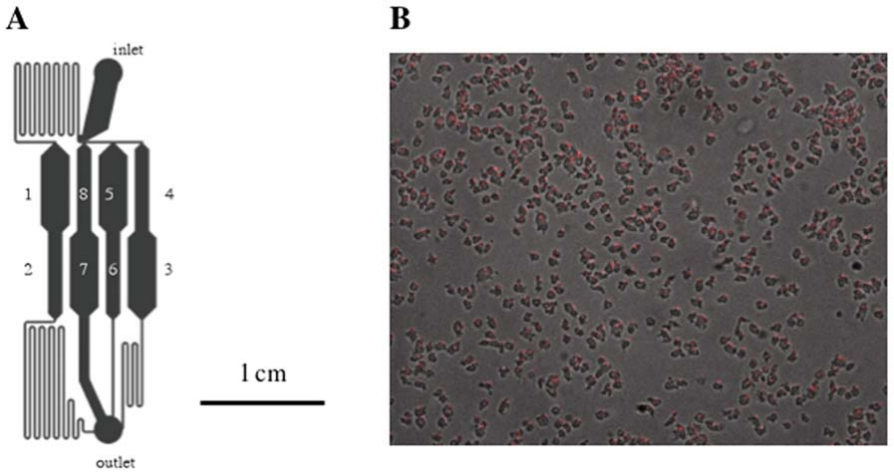
Microfluidic adhesion assay. **A**. Eight chambers and their interconnecting channels were constructed by soft lithography in 5 mm high blocks of PDMS that could be held on cover slips by vacuum. **B.** Cells were spread on cover slips such that approximately 500 cells were initially found in each chamber. Buffer was introduced into the inlet at a pressure of 30 inches water and the chambers imaged every 2 minutes. The number of remaining cells was automatically recorded at each time and normalized to the initial number of cells in the chamber. The flow rate was least in chamber #1 and doubled in each succeeding chamber.

**Figure 2:**
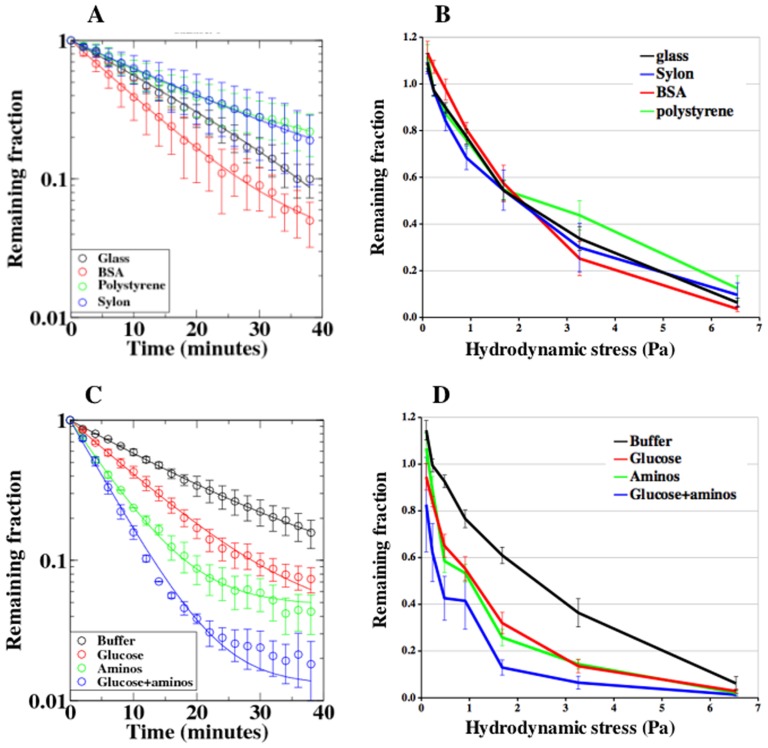
Adhesion to different surfaces. **A.** The kinetics of detachment of cells in chamber #8 (6.5 Pa) was followed for 40 minutes. The remaining fraction of cells is presented on a log scale to show the first order kinetics of detachment from untreated glass (black), Sylon glass (blue), BSA glass (red) and polystyrene (green). Average of at least 5 independent experiments. The bars indicate the S.D. **B**. The remaining fraction of cells after 40 minutes in chambers 2–8 is shown for cells on untreated glass (black), Sylon glass (blue), BSA glass (red) and polystyrene (green). Average of at least 5 independent experiments. The bars indicate the S.E.M. **C**. The effects of glucose (50 mM), amino acids, and both glucose and amino acids of the kinetics of detachment in chamber #8. Average of at least 10 independent experiments. The bars indicate the S.D. **D.** The effects of glucose (50 mM), amino acids, and both glucose and amino acids on the fraction of cells remaining after 40 minutes on glass in the different chambers. Average of at least 10 independent experiments. The bars indicate the S.E.M.

To quantitatively measure cell-substratum adhesion we designed a 1×2 cm microfluidic device with 8 chambers connected with varying resistance to the outlet to generate a range of hydrodynamic shear stresses in a simple but reproducible manner (Fig. 1). The arrangement of channels and chambers ensures that the flow rate doubles from one chamber to the next with the lowest rate in chamber 1 and the highest rate in chamber 8, thereby generating a 128 fold range in hydrodynamic shear stress. Approximately 500 cells were positioned in each chamber and automatically counted every two minutes during 40 minutes of shear stress (Fig. 1B). Cells detached in chamber 8 at 6.5 Pa with approximately first order kinetics consistent with independent responses of the cells (Fig. 2A; Table 1). We filmed cells at higher magnification (63× objective) before and after initiating flow in chamber 8 (see Movies S1 and S2 in Supplementary Materials). Cells can be seen to detach from one frame to the next (<1 second) with no advanced notice. There was no correlation of detachment with changes in cell shape. Even at this high magnification no extracellular material was observed where a cell had just detached.

**Table 1:** Kinetic constants.

Substrate	Ratio of kinetic constants* T_substrate_/T_glass_
BSA coated glass	0.56
Sylon treated glass	1.1
Polystyrene	1.0

Addition	Ratio of kinetic constants T_addition_/T_glass_
Glucose	0.62
Amino acids	0.38
Glucose and Amino acids	0.28

* The rate of cell detachment at 6.5 Pa from different substrates as well as from glass with buffer containing chemical determinants similar to those in the cell membrane were fit with the first order kinetic equation 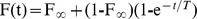. The ratio of the kinetic constant T for the experimental condition to the kinetic constant T_glass_ of control cells on glass is a measure of cell substratum adhesion. Conditions that resulted in more rapid detachment had smaller kinetic constants.

The rates of dissociation from glass, BSA glass, Sylon glass and polystyrene were almost identical. Likewise, the fraction of cells remaining in the different chambers after 40 minutes indicated that the rate of detachment is not affected by the chemical nature or hydrophilicity of the surface (Fig. 2B).

### Interference with substrate adhesion

Small soluble molecules can interfere with van der Waals attraction between closely apposed surfaces if their dielectric properties are similar to those of the surfaces and they are present at concentrations similar to those of the bound adhesive [17]. Since cell surface glycoproteins are not in solution, estimating their effective concentration requires several assumptions. If we consider only the volume where van der Waals attraction forces are strong, then the estimated density of surface glycoproteins in a 30 nm by 1 um^2^ volume is in the range of 10–100 mM for a cell with 5×10^6^ glycoprotein molecules on its surface. Therefore, we tested glucose at 50 mM and a mixture to 12 amino acids at 15 mM (see Methods).

**Figure 3:**
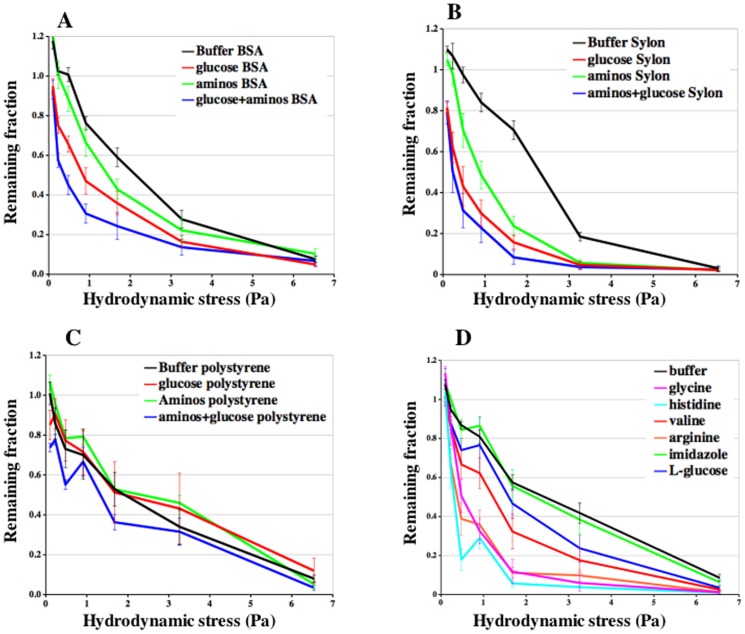
Inhibition of adhesion on different surfaces. The effects of glucose (red), amino acids (green), and both glucose and amino acids (blue) on the fraction of cells remaining after 40 minutes on **A**. BSA glass, **B**. Sylon glass and **C**. polystyrene. **D.** L-glucose, arginine, valine, histidine, glycine and imidazole were added at 50 mM to the adhesion assay buffer. The fraction of cells remaining after 40 minutes in the different chambers were calculated. Average of at least 4 independent experiments. The bars indicate the S.E.M.

The kinetics of detachment at 6.5 Pa was increased two fold by the addition of glucose (Fig. 2C and Table 1). The number of cells remaining after 40 minutes exposure to a wide range of hydrodynamic shear stress was also significantly less when glucose was added to the buffer (Fig. 2D). Likewise, addition of amino acids to the buffer increased the kinetics of detachment and reduced the number of cells remaining at 40 minutes. Addition of both glucose and amino acids further increased the kinetics of detachment and resulted in a greater decrease in the number of cells remaining after 40 minutes (Figure 3A and Table 1). The results suggest that van der Waals attraction between the cells and the glass cover slips is mediated to a large extent by surface glycoproteins.

Glucose and amino acids reduced cell adhesion to the hydrophilic surface of BSA coated coverslips to a similar extent (Fig. 3A) indicating that neither the chemistry nor the surface charge of the substratum determined the nature of the attraction. Adhesion to Sylon treated coverslips was also reduced by addition of glucose or amino acids and more strongly reduced by addition of both glucose and amino acids (Fig. 3B). Addition of either glucose or amino acids had little or no effect on cells sticking to polystyrene although addition of both glucose and amino acids was effective (Fig. 3C). The differences in sensitivity of adhesion on polystyrene to glucose or amino acids suggests that the detailed molecular interactions might be slightly different perhaps as a result of the roughness of the surface of polystyrene.

To determine whether the sugars were acting as a source of nutrients that indirectly affected cell-substratum adhesion, we tested whether the non-metabolizable sugar L-glucose would inhibit adhesion. It turned out to be just as effective as D-glucose (Fig. 3D).

To better define the inhibition by amino acids we tested valine, arginine, histidine and glycine individually at 50 mM and found them to be as effective as the mixture of 12 amino acids (Fig. 3D). Addition of 25 mM or 100 mM valine was found to be slightly less inhibitory than 50 mM valine (data not shown). To further define the critical aspect of the amino acids we tested imidazole, which is the side group on histidine. Addition of 50 mM imidazole had no effect on substratum adhesion (Fig. 3D). Increasing the concentration to 100 mM made no significant difference, ruling out any simple osmotic effects on cell substratum adhesion. It appears that the amino acid moiety, but not the side group, is critical for inhibition of the attractive forces between cells and glass.

**Figure 4:**
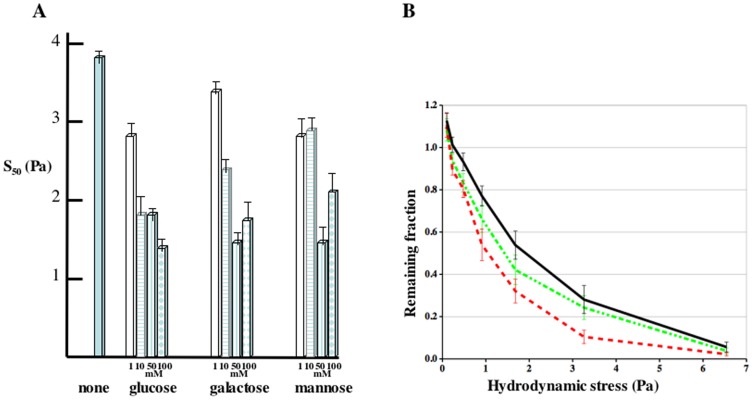
Concentration dependence for sugar inhibition of adhesion. **A.** The shear stress necessary to detach half the cells (S_50_) in buffer, glucose, galactose, or mannose from glass was determined at 40 minutes. Average of at least 4 independent experiments. The bars indicate the S.E.M. **B.** Enzymatic modification of adhesion. Cells were treated with 1 unit of aλπηα-mannosidase (red dashed line) or beta-galactosidase (green dotted line) for 30 minutes and their adhesion to glass compared to that of untreated cells (solid black line). The fraction of cells remaining after 40 minutes in the different chambers were calculated. Average of at least 5 independent experiments. The bars indicate the S.E.M.

Although glycoproteins on the surface of *Dictyostelium* cells are known to carry N-linked high mannose oligosaccharides of 8 to 13 sugars that do not include glucose or galactose [20], inhibition of van der Waals attraction is not expected to be specific to a given sugar. The determinant should be the number of hydroxyl moieties. We tested glucose, mannose and galactose over a range of concentrations in the adhesion assay. Each hexose was equally effective at inhibiting cell substratum adhesion and gave maximal inhibition at 50 mM (Fig. 4A). The lack of specificity rules out interference with a lectin-like receptor. Increasing the concentration of these sugars to 100 mM did not increase the degree of inhibition of adhesion significantly and even reduced the effectiveness of mannose, thereby ruling out the possibility that osmotic effects were involved in reducing cell adhesion.

### Enzymatic modification of the cell surface

If the high mannose oligosaccharides could be removed from the surface glycoproteins, the cells might show reduced substratum adhesion. While it is challenging to quantitatively hydrolyse N-linked oligosaccharide from intact cells, we might be able to enzymatically remove a sufficient proportion to affect adhesion. Cells were incubated with either 1 unit or 10 units of commercially available αlpha-mannosidase or beta-galactosidase for half an hour before being assayed for adhesion to various substrates. Adhesion was reduced in the cells treated with 1 unit αlpha-mannosidase (Fig. 4B). Addition of 10 fold more enzyme made no significant difference (data not shown). Since there are no beta-galactoside linkages in the carbohydrate modifications of *Dictyostelium* proteins [20], cells treated with beta-galactosidase can be considered as controls.

## Discussion

In addition to possibly affecting van der Waals attraction of surface glycoproteins, carbohydrates and amino acids interact with other molecules primarily through hydrogen bonds and ionic forces. It is not unreasonable to consider that hydrogen bonds formed between cell surface components and either untreated glass or BSA coated glass might be disrupted by free sugars or amino acids, but hydrogen bonds are not expected to form to Sylon treated glass or polystyrene. Cells might adhere to these surfaces by interaction of the non-polar face of carbohydrate rings on surface glycoproteins and the vinyl rings of polystyrene or the dimethylchlorosylane groups on Sylon treated glass that could be affected by monosaccharides. However, it is not clear how 50 mM glycine or arginine would disrupt such hydrophobic interactions. Ionic bonds might form between cell surface components and BSA coated glass that could be disrupted by free sugars or amino acids, but such bonds would not be expected to form to untreated glass, Sylon treated glass, or polystyrene. Since adhesion of Dictyostelium cells to each of these substrates is reduced by addition of either glucose or glycine, adhesion to hydrophilic and hydrophobic surfaces would have to be mediated by quite different mechanisms, each sensitive to sugars and amino acids. The data do not unequivocally establish that van der Waals interactions underlie cell substratum adhesion, but they point that way. We do not know the detailed molecular interactions that generate the forces and so cannot tell whether they arise from a permanent dipole and an induced dipole or from instantaneous dipoles. In fact, they may be the sum of many different, rapidly changing interactions. Innate adhesion of cells does not preclude also having specific receptor-ligand mechanisms of adhesion. Integrin mediated focal adhesions of mammalian cells generate much stronger adhesion to extracellular matrices than the forces holding *Dictyostelium* cells to untreated glass and can be useful when invading crowded environments [2], [14]. While the innate adhesion of *Dictyostelium* is sufficient to provide traction for rapid movement on a variety of substrata, several mutant studies have implicated specific proteins in cell-substratum adhesion. They include the F-actin binding protein, talin, the nine transmembrane proteins, Phg1A and SadA, and a large protein that carries a type A von Willebrand factor domain, SibA [21]–[26]. Most of these are intracellular proteins that are unlikely to be ligand specific adhesion proteins, but their mutant phenotypes suggest that the cytoskeleton plays a role in substratum adhesion. Nevertheless, SibA has been proposed to act in a manner similar to integrins [25]. While the sequence of SibA is unrelated to that of any integrin, SibA and integrins can both bind to talin. However, it is unclear whether it acts in a manner similar to integrins, which function as obligate heterodimers and recognize the three amino acid motif RGD in fibronectin and other proteins [2], [7]. No partner for SibA has been found in *Dictyostelium* and the relevant extracellular matrix components are missing.

Since *Dictyostelium* cells can rapidly and reversibly stick to a wide variety of substrates of differing chemical composition and hydrophobicity, adhesion does not appear to depend on ligand binding, covalent, hydrophobic or ionic bonds, leaving van der Waals attraction as the most likely mechanism of adhesion. Regions that are close to each other can form induced dipoles that attract each other such that almost any surface will be attracted to any other where they come into close apposition [17]. For instance, geckos can hold their full weight on a vertical glass surface by the combined van der Waals attraction forces of millions of 200 nm wide spatula structures on the thousands of setae that cover their foot pads [27]. By close apposition of a large area these lizards can even cross a glass ceiling.

Since cell surfaces have glycoproteins protruding all over them, the first molecules to be closely juxtaposed to the substratum will include the glycoproteins. Experimental determination of the parameters affecting van der Waals attraction have shown that sugars can generate more interactive energy than proteins or phospholipids [17]. Sugars in general make good adhesives as shown by the effect of coating gecko-inspired silicon based dry adhesives with polymers that are chemically similar to the oligosaccharides on glycoproteins [28], [29]. Only after such structures are coated with the glycopolymers are they strongly adhesive under water. Since glycosylation of membrane proteins is widespread in all cell types, it is a universal feature of their surfaces that could be used for adhesive interactions.

Further evidence that sugar moieties on the surface of cells are involved in adhesion comes from the demonstration that glucose, galactose, and mannose are each able to significantly reduce adhesion when added at 50 mM to the buffer that flows through the microfluidic adhesion assay (Figs. 3, 4). Moreover, 50 mM glucose inhibits adhesion to naked glass, BSA coated glass, and Sylon treated glass suggesting that sugars are participating in generating the adhesive force to these radically different surfaces. Importantly, cells treated for half an hour with αlpha-mannosidase just prior to measurement of substratum adhesion showed significantly reduced substratum adhesion (Fig. 4).

Addition of a mixture of 12 amino acids, each at about 2 mM, was also effective at reducing adhesion to naked glass, BSA coated glass, and Sylon treated glass. These results suggest that the protein portions of surface glycoproteins are also involved in forming molecular interactions with the various surfaces. Individual amino acids (arginine, valine, histidine, glycine) are able to inhibit cell substratum adhesion at 50 mM (Fig. 3D). The fact that histidine inhibits, while its side group imidazole does not, indicates that the target is the peptide backbone of surface proteins. Furthemore, the underivatized amino acid glycine is an effective inhibitor. Just as glycoproteins are prevalent on the surface of almost every animal cell, all cells have proteins on their surface that would be expected to be able to generate non-specific innate adhesion by van der Waals attraction of the peptide backbone and the substratum providing traction without the need for specialized adhesion mechanisms.

Our results are also compatible with a slightly more complicated mechanism of cell-substrate adhesion in which the cells first secrete a polymer which sticks strongly to the substratum and then they adhere to it. Since the cells are adhesive to clean glass, hydrophilic BSA treated glass, hydrophobic Sylon treated glass, and hydrophobic polystyrene, the hypothetical “red carpet” polymer must also be able to rapidly adhere to these surfaces. van der Waals attractions are a likely means for adhesion of the polymer to such diverse substrata. The polymer would also have to be rapidly secreted since cells can be observed to adhere to these substrata within a minute even in the constant flow of buffer in the microfluidic devices. The only real difference between such a mechanism and the direct van der Waals attraction between surface glycoproteins and the substratum is that the adhesive carpet would be left behind. Although we see no evidence for trails behind motile cells, such trails might be difficult to visualize. Evidence for secreted material essential for cell-substratum adhesion might come from further characterization of mutant strains selected for loss of substrate adhesion. Genes that are essential for secretion of the adhesive polymer or those encoding cell surface receptors necessary for binding to the “red carpet” could provide biochemical support for such a mechanism.

Since traction depends on coupling the adherent regions of the cell surface to the underlying cytoskeletal cortex, any gene that encodes a component essential for such coupling would turn up in a screen for slow or non-motile mutant cells. An extensive catalog of such genes, along with a better understanding of the mechanism of cell-substrate adhesion, would make significant progress towards understand both basal and chemotactic motility.

## Methods

### Cells and chemicals

Strain AX4 transformed with a construct kindly provided by Robert Cooper and Ted Cox, Princeton University, in which Red Fluorecent Protein (RFP) with a nuclear localization peptide driven by the actin 15 promoter was used in all experiments. Fresh inocula were prepared from lyophilized stocks every few weeks and used in experiments for up to a month. Since we found innate substratum adhesion to be quite sensitive to prior growth conditions, we worked exclusively with cells growing exponentially in suspension in filter sterilized HL5 medium that had not exceeded a density of 2×10^6^ cells/ml. Cells in the exponential phase of growth were deposited on plastic petri dishes in HL5 for 18 hours to allow multinucleated cells to divide to cells of uniform size before measurement of substratum adhesion in a microfluidic device (Fig. 1). Cells were washed and suspended in 20 mM sodium potassium phosphate buffer pH 6.4 with 200 uM calcium at 5×10^5^ cells/ml. A drop of the suspension was placed on a cover slip and the device lowered over them in the absence of flow and held by vacuum. The cells were allowed to attach for 5 min during which time many cells could be seen to crawl around before buffer was allowed to flow through the chambers. The flow rate was adjusted so that almost no cells were detached over 40 minutes in the chambers with the least flow, while most cells were dislodged in the two chambers with the fastest flow. Approximately 500 cells were present in each of the chambers at the start. Each experiment included a control of cells in buffer on untreated glass. Cells from the same population were then tested with other substrata in the presence or absence of small molecules and their adhesion compared to that of the control.

Unless otherwise stated all chemical and media components were purchased from Fisher Scientific. D-mannose was purchased from Sigma. L-glucose was purchased from Invitrogen. L-amino acids were purchased from Sigma. Sylon [5% dimethyldichlorosilane in toluene] was purchased from Sigma.

The mixture of amino acids that was used to inhibit cell-substratum adhesion contained 3 mM L-arginine, 0.5 mM L-cystine, 1 mM L-histidine, 2 mM L-isoleucine, 1.8 mM L-leucine, 2.5 mM L-lysine, 0.5 mM L-methionine, 1 mM L-threonine, 0.25 mML-tryptophan, 1 mM L-tyrosine, and 2 mM L-valine. The mixture was purchased from Mediatech.

### Microfluidics

1×2 cm microfluidic devices with 8 chambers connected with varying resistance to the outlet were formed in silicon elastomer PDMS by soft lithography (Figure 1). The arrangement of channels and chambers ensured that the flow rate doubled from one chamber to the next generating a 128 fold range in hydrodynamic shear. The input pressure was set at 30 inches water resulting in a flow rate of ∼18 mm/sec in chamber 5, determined by following fluorescent beads added to the input buffer. To load approximately equal numbers of cells into each chamber, we spread cells evenly on a coverslip and then placed the device over them. The number of cells in each chamber was determined by fluorescence microscopy using a 10× objective. The stage moved every 100 ms to a new chamber and automatically refocused. The cycle was repeated every 2 minutes. The number of cells in each frame was automatically recorded and the proportion remaining was calculated relative to the starting number in each chamber. The reliability of automatic counting was greatly improved by using cells that express Red Fluorescent Protein (RFP) carrying a nuclear localization signal that makes all the nuclei fluoresce in the red but has no other observable effects. The separation of nuclei ensures that two adjacent cells are not confused for a single cell. The number of cells in the first few chambers was often seen to increase when the flow was initiated probably as the result of cells being washed down from the upstream resistence channels and settling in the low flow chambers (Fig. 1). We consider chamber #1 to be a settling pond and did not include it in subsequent analyses. Half the cells detached from glass when the pressure was about 2.5 Pa, the same pressure that was found in the radial flow detachment assay to detach half the cells [14].

Borosilicate glass cover slips were coated with bovine serum albumin (BSA) by immersing them in a 0.2% solution of BSA for 20 minutes and then allowing them to dry in air. Cover slips were rendered hydrophobic by soaking them in Sylon and then allowing them to dry in air. Polystyrene substrata were prepared by breaking the covers of plastic petri dishes into the appropriate size. Since the manufacturers were not particularily concerned that the petri dish lids be uniformly flat, we encountered problems in focusing on certain chambers. This resulted in somewhat more noisy data for adhesion to polystyrene. Moreover, the surface of polystyrene is rougher than glass.

### Fitting procedure

To determine the kinetics of detachment in chamber #8, we first averaged the experimental data obtained in each experiment and determined the fraction of non-detached cells, F, computed with respect to the number of attached cells at time point t = 2 min, as a function of time. This average was then fitted using the formula 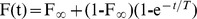.

The two fitting parameters are T, the time constant of detachement, and F_∞_, the fraction of cells that remain attached at infinite time. This fit was motivated by earlier work that suggested that a significant fraction of cells will remain indefinitely attached [14]. We also used a fit using a simple single exponential, containing only a single fitting parameter T. Our fitting results revealed that the two parameter fit gives a slightly better result, as determined using the Akaike's information criterion, a measure of the relative goodness of fit. However, both fits give almost identical time constants while F_∞_ is close to zero in most cases.

### Measurement of hydrophobicity

Hydrophobicity of the surfaces was determined by measuring the wetting angles of droplets of distilled water on the various substrata. On the most hydrophobic surfaces droplets approach hemispherical forms with wetting angles of about 90° while they spread on the most hydrophilic surfaces such that the wetting angle is less than 1°. We found that clean glass gave a wetting angle of 38°, Sylon treated glass gave 98°, polystyrene gave 85°, and BSA coated glass gave <1°.

## Supporting Information

Movie S1 **Cells in chamber 8 of the microfluidic device were imaged by DIC using a 63× objective every second starting 30 seconds before the flow was initiated.** Hydrodynamic shear stress of 6.5 Pa was generated in 20 mM sodium potassium phosphate buffer.
(MOV)

Movie S2 **Same as Movie S1 except that 50 mM glucose was present in the buffer.**
(MOV)
